# Role of Genetic Polymorphism of Angiotensin-Converting Enzyme, Plasminogen Activator Inhibitor-1 and Endothelial Nitric Oxide Synthase in the Prognosis of Coronary Artery Disease

**DOI:** 10.4021/cr108e

**Published:** 2010-11-20

**Authors:** Ai Yuan Zhang, Xiang Wu Ji, Ai Juan Zhang, Li Xue Guan, Jing Huang, Jing Xian Wang

**Affiliations:** a Department of Cardiology, Affiliated Wei Fang People’s Hospital of Wei Fang Medical College, Wei Fang, Shandong Province, China

**Keywords:** Coronary artery disease, Gene polymorphism, Angiotensin-converting enzyme, Endothelial nitric oxide synthase, Plasminogen activator inhibitor-1, Prognosis, Major adverse cardiovascular event

## Abstract

**Background:**

This study was to investigate the effects of multiple genetic polymorphisms and conventional risk factors in the prognosis of coronary artery disease (CAD).

**Methods:**

One hundred and fifty five patients with CAD were prospectively recruited, they were subgrouped as single vessel disease (SVD) and multiple vessel disease (MVD). All patients were detected I/D polymorphism of angiotensin-converting enzyme (ACE) gene, 4G/5G polymorphism of plasminogen activator inhibitor-1 (PAI-1) gene, and G894→T mutation of endothelial nitric oxide synthase (eNOS) gene. The patients were followed up for 10-65 months, mean 35 months. End points were major adverse cardiovascular events (MACE), including angina, myocardial infarction, and cardiac sudden death.

**Results:**

During the follow-up period, MACE developed in 81 patients, 73 patients with angina, seven with myocardial infarction, and one with cardiac sudden death. CAD patients with MVD were more probable of developing MACE during follow-up. Distribution of PAI-1 gene polymorphism was significantly different between SVD and MVD patients, p < 0.001. The frequency of DD genotype of ACE and 4G/4G genotype of PAI-1 in patients with MACE were significantly higher than those in patients without MACE, p < 0.001 and p = 0.002, respectively. Incidence of diabetes mellitus was significantly higher in patients with MACE than in patients without MACE, P = 0.03. Cox regression analysis showed that diabetes mellitus (HR 2.36, 95% CI 1.33-4.46, p = 0.003), 4G/4G polymorphism of PAI-1 gene (HR 3.45, 95% CI 1.71-6.56, p = 0.009), and D/D polymorphism of ACE gene (HR 2.99, 95% CI 1.84-5.76, p = 0.005), were independent predictors of the MACE.

**Conclusions:**

Our results showed that the conventional risk factors and genetic polymorphisms have significant influence on prognosis of CAD patients. CAD patients with diabetes mellitus, DD genotype of ACE, and 4G/4G genotype of PAI-1 suggested poor prognosis.

## Introduction

Coronary artery disease (CAD) can be caused by multiple factors from conventional and genetical aspects, and these factors are interactive [1-4]. Several genetic systems are involved in the pathophysiology of CAD, among these, angiotensin I-converting enzyme (ACE) plays role in modulating vascular tone and electrolyte balance by hydrolyzing angiotensin I to angiotensin II, which is a potent vasopressor and aldosterone-stimulating peptide [5], the individual variation of plasma ACE levels is largely affected by polymorphism of the ACE gene. Previous studies showed that the I/D polymorphism of ACE is highly related to the coronary artery disease (CAD), the DD genetic type is the independent risk factor of CAD [6-8].

It has been shown that endothelial nitric oxide synthase (eNOS) inhibition can accelerate atherosclerosis in animal models, the abnormalities in the eNOS pathway are present in humans with atherosclerosis [9]. Therefore, functional variants of the eNOS gene could influence individual susceptibility to atherosclerosis by altering the amount of NO generated by the endothelium. Among the several polymorphisms of the eNOS gene, the common variant located in exon 7 (G894→T) that modifies its coding sequence (Glu298→Asp) has been linked to the risk for coronary artery spasm, and acute myocardial infarction (AMI) [10, 11].

The plasminogen activator inhibitor-1(PAI-1) 4G/5G polymorphism is also related to CAD, the carriers of 4G/4G allele have high incidence of CAD [12], they are susceptible to CAD under the same conventional factors.

The interactive effects of aforementioned genes polymorphism on the clinical features and prognosis of CAD are not fully elucidated. Studies including these three important genes polymorphism encompassing three pathophysiological pathways on CAD severity and prognosis are rare. In this study, we investigated the prevalence and interaction of these pathway genes polymorphims, and their interaction with conventional risk factors in the severity and prognosis of CAD in Chinese population.

## Materials and Methods

### Study population

Study subjects were prospectively recruited from April 2001 to November 2005, all patients were diagnosed as CAD by quantitative coronary angiography, with more than 50% luminal diameter stenosis affecting at least one coronary vessel.

The study subjects were stratified into two subgroups according to the number of diseased major vessels (left anterior descending, left circumflex, and right coronary artery and their major branches) as single vessel disease (SVD) and multiple vessel disease (MVD) which affected more than one vessels. If only the left main coronary was affected, it was recorded as MVD.

Medical histories of hypertension, diabetes mellitus and cigarette smoking were obtained. Hypertension was diagnosed when elevated blood pressure (> 140/90 mmHg) was measured on 3 occasions or were already being treated with antihypertensive agents. Diabetes mellitus was defined as having a fasting blood glucose level of > 7.0 mmol/L on at least two separate occasions, or was already receiving treatment for diabetes. Subjects with cardiomyopathy, congenital heart disease, renal or hepatic disease, valvular disease, were excluded from the study. One hundred and ninety healthy controls were recruited consecutively from those who were admitted to our hospital for routine health examination during the same period. These controls had no histories of coronary artery disease, cerebrovascular disease or peripheral arterial disease.

All subjects in study and control groups were from the Chinese Han population, they were genetically unrelated. This study protocol was approved by the bioethical committee of Weifang people’s hospital, and was in accordance with the Declaration of Helsinki. Informed consent was obtained from every participant.

### Genotyping

Genomic DNA was prepared from samples of whole blood by standard methods. Peripheral blood leukocyte DNA was extracted, genotypes were assessed by PCR-based method.

PCR assay for I/D polymorphism of the ACE gene was performed as previously described [13], 0.1 lag genomic DNA was amplified with a forward primer (5'-CTGCAGACCACTCCCATCCTTTCT-3'), a reverse primer (5'-GATGTGGCCATCACATTCGTCAGAT-3').

PCR assay for Glu298-Asp polymorphism in exon 7 of Enos was performed as previoiusly described [10], 0.1 lag genomic DNA was amplified with a forward primer (5'-CATGAGGCTCAGCCCCAGAAC-3'), a reverse primer (5'-AGTCAATCCCTTTGGTGCTCAC-3').

PCR assay for 4G/5G polymorphism of the PAI-1 gene was performed as previoiusly described [14, 15], 0.1 lag genomic DNA was amplified with a forward primer (5'-CAC AGA GAG AGT CTG GCC ACGT -3'), a reverse primer (5'-CAG CCA CTG GAT TGT CTA GGT-3').

Amplification was performed in a final volume of 25 µl containing 0.75 µmol of each primer, 1.2 mmol/L MgC1_2_, 50 mmol/L KCl, 10 mmol/L Tris HC1, pH 8.3, 0.001% gelatin, 5% dimethylsulfoxide, 0.24 mmol/L of each dNTR and 1 U Taq polymerase. DNA amplification was achieved by an initial denaturation at 94 °C for 5 minutes, followed by 30 cycles with denaturation at 94 °C for 1 minute, annealing at 60 °C for 30 seconds, and extension at 72 °C for 30 seconds, and then final extension at 72 °C for 5 minutes. PCR products were subjected to 6% agarose gel electrophoresis.

### Blood biochemistry

Blood samples were collected 6 hours after fasting, total cholesterol, triglycerides, glucose were measured using CX-9 automatic biochemistry analyzer (Beckman, Fullerton, USA ).

### Follow-up

All patients received usual therapy for CAD based on the decision of the cardiologists in charge of the patients. Follow-up was conducted by means of telephone interview, review of medical records, and/or report from the investigators. This study cohort was followed until November 2006, end points were major adverse cardiovascular events (MACE), which include angina, myocardial infarction and cardiac sudden death. Diagnosis of myocardial infarction was based on ischemic chest symptoms, typical electrocardiographic changes, and elevation of serum creatine kinase. All events were documented on hospital records, investigator reports, and/or patients or family information.

### Statistics

Data of age and biochemical variables were presented with mean value ± SD, and differences were compared by Student’s *t* test. Differences among the gene polymorphisms were analyzed by Chi-square test or Fisher’s exact test wherever appropriate. The probability of MACE-free survival was estimated by Kaplan-Meier method, log-rank test was used to compare the MACE-free survival between SVD and MVD subtypes. Univariate and multivariate estimates of hazard ratios (HR) were calculated using the Cox regression analysis. Statistical analysis was carried out using SPSS statistical program. A value of P <0.05 value was considered significant.

## Results

### Baseline demographic and clinical characteristics

During the study period, 155 patients with angiographically defined CAD were included, mean age 63.7 ± 9.8 years, median 67 yeas, range 25 to 79 years. Fifty-four patients were diagnosed as SVD, 101 patients as MVD, table 1 shows the prevalence of vascular risk factors and genetic polymorphisms in patients and controls. The CAD patients had higher prevalence of hypertension, diabetes mellitus, smoking, total cholesterol and triglycerides, compared to that in the control group.

**Table 1:** Baseline Demographic and Clinical Characteristics

	CAD patients	Control	P value
	n (%)	n (%)	
No. of subjects	155	190	
Age(year, mean±SD)	63.7 ± 9.8	63.3 ± 9.4	0.7
Male/famale	113/42	110/80	0.005
Diabetes	35 (22.5)	10 (5.2)	< 0.001
Hypertension	72 (46.4)	21 (11)	< 0.001
Smoking	52 (33.5)	55 (28.9)	0.04
TC (mmol/L)	4.93 ± 1.06	3.6 ± 1.3	< 0.001
TG (mmol/L)	1.83 ± 1.25	1.5 ± 1.2	0.024
PAI-1 gene (4G/5G)			
5G5G	35 (22.5)	61 (32,1)	0.006
4G5G	62 (40)	87 (45.8)	
4G4G	58 (37.5)	42 (22.1)	
ACE gene (I/D)			
			
II	49 (31.6)	93 (48.9)	0.002
ID	56 (36.1)	61 (32.1)	
DD	50 (32.3)	36 (19)	
eNOS gene			
GG	113 (72.9)	156 (82.1)	0.029
GT	36 (23.3)	33 (17.4)	
TT	6 (3.8)	1 (0.5)	

TC, total cholesterol; TG, triglycerides

Significant difference was observed in the distribution of the PAI-1, ACE and eNOS genetic polymorphism between patients and controls, p = 0.006, p = 0.002 and p = 0.029, respectively.

### Risk factors and genetic polymorphisms in SVD and MVD

Distribution of conventional risk factors and genetic polymorphisms in SVD and MVD patients is shown in table 2. The prevalence of diabetes mellitus in MVD group was significantly higher than that in SVD group. There was no significant difference in the other conventional factors between SVD and MVD group (p = 0.003). There was significant difference in the distribution of PAI-1 gene polymorphism between SVD and MVD patients (p < 0.001).

**Table 2:** Baseline Clinical Characteristics and Gene Polymorphisms Between Different CAD Subtypes

	SVD	MVD	P value
	n (%)	n (%)	
No. of subjects	54	101	
Age (year, mean ± SD)	61.7 ± 11.8	64.3 ± 8.4	0.11
Male/famale	40/14	73/28	0.85
Diabetes	11 (20.3)	46 (45.5)	0.003
Hypertension	25 (46.3)	47 (46.5)	1.00
Smoking	19 (35.2)	33 (32.7)	0.85
TC (mmol/L)	4.8 ± 1.9	4.6 ± 1.2	0.31
TG (mmol/L)	1.93 ± 1.1	1.7 ± 0.9	0.16
PAI-1 gene (4G/5G)			
5G5G	22 (40.7)	13 (12.9)	< 0.001
4G5G	21 (38.9)	41 (40.6)	
4G4G	11 (20.4)	47 (46.5)	
ACE gene (I/D)			
II	17 (31.5)	32 (31.7)	0.08
ID	14 (25.9)	42 (41.6)	
DD	23 (42.6)	27 (26.7)	
eNOS gene (G/T)			
GG	44 (81.5)	69 (68.3)	0.09
GT+TT	10 (18.5)	32 (31.7)	

SVD, single vessel disease; MVD, multiple vessel disease; TC, total cholesterol; TG, triglycerides.

### MACE in SVD and MVD

**Figure 1:**
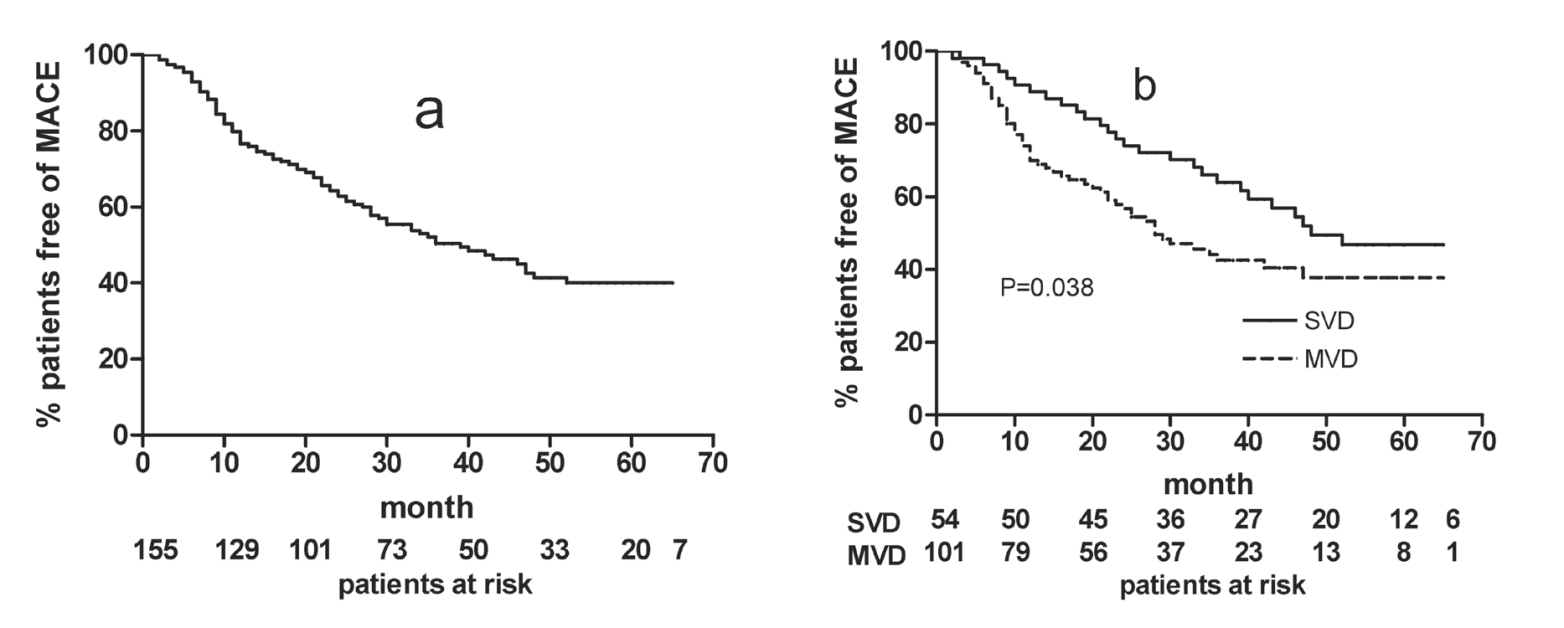
Kaplan-Meier analysis for probability of MACE free patients. (a) Probablility of MACE free survival in all patients. (b) Probabilities of MACE survival in SVD and MVD patients, p=0.038, log-rank test.

The 155 CAD patients were followed up for a period of 10-65 months, the mean follow-up duration was 38 ± 17.2 months, median 36.5 months. MACE occurred in 81 patients, with 73 angina, seven MI, and one cardiac sudden death (Table 3). Kaplan-Meier analyis was conducted, the probability of MACE free in all patients is shown in figure 1a, the probabilities of MACE free patients in SVD and MVD are shown in figure 1b. Log-rank test showed that there was significant difference in MACE free probability between SVD and MVD group, p = 0.038, this revealed that in the MVD group, there was higher probability of MACE development.

**Table 3:** MACE Results in Different CAD Subtypes During Follow-Up

Follow-up Outcome	SVD (n = 54)	MVD (n = 101)
Angina	24	49
MI	2	5
SCD	0	1

SCD, sudden cardiac death; MI, myocardial infarction.

### Clinical characteristics and gene polymorphisms among patients with and without MACE during follow-up

During follow-up period, patients with diabetes mellitus and smoking showed significant high incidence of MACE, p = 0.03 and p = 0.01 respectively. The distribution of PAI-1 gene (4G/5G) and ACE gene (I/D) showed significant differences between patients with and without MACE, p < 0.001 and p = 0.002, respectively, Table 4.

**Table 4:** Clinical Characteristics and Gene Polymorphisms Among Patients With and Without MACE During Follow-up

	With MACE	Without MACE	P value
	n (%)	n (%)	
No. of subjects	81	74	
Age (year, mean±SD)	62.1 ± 10.8	63.1 ± 9.1	0.53
Male/famale	61/20	52/22	0.59
Smoking	32 (39.5)	20 (27)	0.01
Diabetes mellitus	24 (29.6)	11 (14.9)	0.03
Hypertension	39 (48.1)	33 (44.6)	0.75
TC (mmol/L)	5.2 ± 1.4	4.8 ± 1.2	0.06
TG (mmol/L)	1.9 ± 1.3	1.8 ± 1.5	0.65
PAI-1 gene (4G/5G)			
5G5G	10 (12.3)	25 (33.8)	< 0.001
4G5G	31 (38.3)	31 (41.9)	
4G4G	40 (49.4)	18 (24.3)	
ACE gene (I/D)			
II	19 (23.4)	30 (40.5)	0.002
ID	26 (32.1)	30 (40.5)	
DD	36 (44.5)	14 (19)	
Enos gene (G/T)			
GG	57 (70.4)	56 (75.7)	0.48
GT+TT	24 (29.6)	18 (24.3)	

MACE, major adverse cardiovascular event; TC, total cholesterol; TG, triglycerides

### Cox univariate analysis

Table 5 summarizes the results of Cox univariate analysis. The hazard ratio of male gender was 1.22 compared with female gender, and was statistically significant, p = 0.014. The hazard ratio in patiens with diabetes mellitus or hypertension was also statistically significant compared with those without diabetes mellitus or hypertension, p = 0.002 and p = 0.01, respectively. The hazard ratio of 4G/4G polymorphism of PAI-1 was 3.54 compared with that of 5G/5G, p < 0.001. The hazard ratio of DD polymorphism of ACE gene was 2.86 compared with that of II, p = 0.003.

**Table 5:** Univariate Hazard Ratios by Cox Regression Analysis for the MACE

Variables	Hazard ratio	95% CI	P value
Age			
≤60	1		
>60	1.12	0.82 - 3.22	0.36
Male gender			
Female	1		
Male	1.22	1.02 - 2.28	0.014
Diabetes mellitus			
No	1		
Yes	2.57	1.39 - 5.66	0.002
Hypertension			
No	1		
Yes	1.95	1.28 - 3.97	0.01
Smoking			
No	1		
Yes	0.89	0.32 - 1.86	0.79
TC			
≤5.7	1		
>5.7	1.23	0.47-2.76	0.81
TG			
≤1.7	1		
>1.7	1.77	0.63 - 3.99	0.57
PAI-1 gene			
5G/5G	1		
4G/5G	1.4	0.98 - 2.41	0.43
4G/4G	3.54	1.24 - 7.33	<0.001
ACE gene			
II	1		
I/D	1.9	0.76 - 3.1	0.32
DD	2.86	1.64 - 5.89	0.003
eNOS gene			
GG	1		
GT+TT	1.28	0.34 - 2.45	0.17

### Cox multivariate analysis

From the results of univariate analysis, the factors of male gender, diabetes mellitus, hypertension, 4G/4G polymorphism of PAI-1 gene, and DD polymorphism of ACE gene were statistically significant, Cox multivariate analysis was carried out for these factors. The results showed that hazard ratios for diabetes mellitus, 4G/4G of PAI-1 gene and DD of ACE gene were high and statistically significant, p = 0.003, p = 0.009, p = 0.005, respectively, these three factors were the independent prognostic factors for MACE in CAD patients (Table 6).

**Table 6:** Multivariate Hazard Ratio by Cox Regression Analysis for the MACE Events

Variables	Hazard ratio	95% CI	P value
Male gender			
Male	1		
Female	0.98	0.85 - 2.38	0.32
Diabetes mellitus			
No	1		
Yes	2.36	1.33 - 4.46	0.003
Hypertension			
No	1		
Yes	1.87	0.98 - 3.88	0.12
PAI-1 gene			
5G5G	1		
4G5G	1.22	0.88 - 2.12	0.51
4G4G	3.45	1.71 - 6.56	0.009
ACE gene			
II	1		
ID	1.82	0.66 - 2.6	0.52
DD	2.99	1.84 - 5.76	0.005

## Discussion

It has been hypothesized that a genetic component can condition the development of CAD, in the present study, we compared the distributions of conventional risk factors and the three genetic polymorphisms (PAI-1, ACE and eNOS) in a cohort of CAD patients according to the follow-up MACE. To our knowledge, there was no such investigation before.

Our results indicated that the conventional cardiovascular risk factors presented in the CAD patients, such as smoking, hypercholesterolemia, hyperglycermia, diabetes mellitus, hypertension, were the most important risk factors in our CAD patients, smoking and diabetes mellitus were significantly associated with MACE development during follow-up.

Previous studies showed that I/D polymorphism of ACE gene was associated with CAD, the DD genotype and D allele are the independent risk factors of CAD [8, 16]. Survival rate of carriers with ACE DD genotype was significantly lower than that of ID and II carriers [17]. However, Larsen reported that the ACE polymorphism was not related with the CAD [18], similar results were also obtained by other researchers [19]. In our study, we found that there was no significant difference of ACE genotype distribution in SVD and MVD, however the MACE occurred more frequent in patients with DD genotype. Due to the enhanced ACE activity by D allele, the production of angio-tensin II is increased which enhances degradation of cardiovascular protective agent bradykinin, this results in the local blood vessel contraction and the hypertrophy of myocardiac muscle and blood vessel smooth muscle, which causes the vessel spasm and instability of coronary artery atherosclerosis plaque, then the unstable angina or MI follows.

The deletion/insertion polymorphism in the promoter region of the PAI-1 gene was identified in 1993 [20]. This polymorphism includes a “deletion” allele with a sequence of four guanosines (4G) and an “insertion” allele with five guanosines (5G) [20]. Studies showed that the 4G/5G gene polymorphism was associated with a significant increase in risk of CAD and MI. Maurizio reported that the cells with 4G/4G homozygous produces more PAI-1 [21]; the 4G allele is associated with the increasing risk of CAD [22], 4G allele carriers have high risk of coronary thrombosis and sudden cardiac death, with high incidence of AMI [23, 24]. However, in other studies, no association of the PAI-I gene variation with the risk of CAD was observed [12, 25]. In our study, we found that preverlence of 4G/4G genotype significantly higher in the MACE patients, and 4G/4G genotype was more frequent in MVD than in SVD.

Several polymorphisms have been identified in the eNOS gene, among which is one located in exon 7 (G894→T) which modifies its coding sequence (Glu^298^→Asp). This variant has been reported to associate with coronary spasm, CAD and acute MI [10, 26]. The homozygous of Glu^298^→Asp polymorphism might be the genetic factor of early MI, but not related with the severity of coronary artery athroserosis [11]. Variation of G894T may be the independent risk factor of coronary in-stent restenosis, the 298ASP allele carriers had higher coronary in-stent restenosis [27, 28]. Wei et al also found the mutation frenquency of eNOS gene G894→T was not significant different among the single vessel, bi-vessel or multiple vessels CAD in Chinese Han race patients diagnosed by coronary angiography, this observation was in aggrement with ours. We found the Glu298→Asp polymorphism of eNOS gene was not associated with MACE and severity of diseased vessels in CAD.

Cox regression multivariate analysis showed that DD polymorphism of ACE gene, 4G/4G polymorphism of PAI-1 and diabetes mellitus are independent prognostic risk factors of CAD. The genetic susceptible individuals can be instructed to control the conventional risk factors in order to prevent or treat the CAD in early stage.

In conclusion, CAD patients carrying DD genotype of ACE gene are susceptible of MACE. CAD patients carrying 4G/4G genotype of PAI-1 gene are susceptible of multiple vessel disease and MACE during follow-up. The DD polymorphism of ACE gene, 4G/4G polymorphism of PAI-1 gene as well as diabetes mellitus, are the independent prognostic factors of CAD.
